# Aneurysm of the Vein of Galen Diagnosed with MRI

**DOI:** 10.1155/2013/716762

**Published:** 2013-02-20

**Authors:** Themistoklis Dagklis, Chrysoula Margioula-Siarkou, Stamatios Petousis, Theodoros Xenidis, Aggelos Sapidis, Ioannis Kalogiannidis, David Rousso

**Affiliations:** 3rd Department of Obstetrics and Gynaecology, Aristotle University of Thessaloniki, Konstantinoupoleos 49, 54642 Thessaloniki, Greece

## Abstract

We describe the case of a neonate with aneurysm of vein of Galen that was diagnosed prenatally in the 33rd gestational week by MRI. A 27-year-old woman, gravida 2, para 2, was admitted to our department at 33 weeks of gestation with suspected fetal hydrocephaly. Ultrasound examination after admission demonstrated an anechoic, supratentorial, and median mass with regular borders, raising the possible diagnosis of an aneurysm of the vein of Galen. MRI confirmed the presence of an aneurysm of the vein of Galen. An elective caesarean section was performed at 33 weeks of gestation. The newborn was admitted to the Neonatal Intensive Care Unit. Despite the full respiratory and medical support given, the sustainment of cardiac failure resulted in neonatal death just one day following its admission.

## 1. Introduction

The aneurysm of the vein of Galen represents a rare intracranial arteriovenous malformation, accounting for 1% of all fetal arteriovenous vascular abnormalities [1]. Mainly correlated with the persistence of the embryonic median prosencephalic vein of Markowski [2], the dilatation of vein of Galen may have a severely detrimental impact on fetal or neonatal heart function, resulting in increased rates of neonatal morbidity and mortality [3]. As the vein of Galen develops multiple connections with cerebral arteries, an increased cardiac preload is produced, leading to severe heart failure as early as in fetal life [4]. The prognosis in cases of vein of Galen aneurysmal malformations (VGAM) is often poor, with quick development of multisystem organ failure despite vigorous management [5]. Recent advances in supportive medical care and innovation such as embolism have helped improve the outcome in cases with VGAM [3]. Early detection is crucial in order to proceed to effective therapeutic management. 

We describe the case of a neonate with aneurysm of vein of Galen that was diagnosed in the 33rd gestational week by MRI.

## 2. Case Report

**Figure 1:**
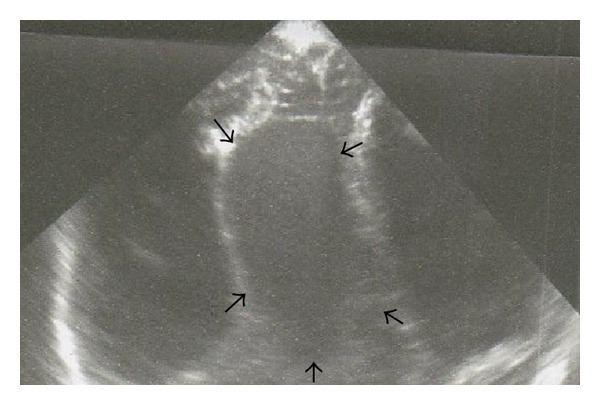
Ultrasound imaging of our case's aneurysm of vein of Galen.

A 27-year-old woman, gravida 2, para 2, was admitted to our department at 33 weeks of gestation with suspected fetal hydrocephaly during her routine third trimester ultrasound. A fetal medicine specialist ultrasound examination at 22 weeks of gestation had not revealed any fetal abnormalities. Ultrasound examination after admission demonstrated an anechoic, supratentorial, and median mass with regular borders, raising the possible diagnosis of an aneurysm of the vein of Galen (Figure 1). Fetal hydrops along with cardiomegaly were also observed. Doppler examination indicated turbulent blood flow of arterial and venous vessels. Blood examinations were performed, and a blood sample was sent to exclude infection by Parvo-19. 

**Figure 2:**
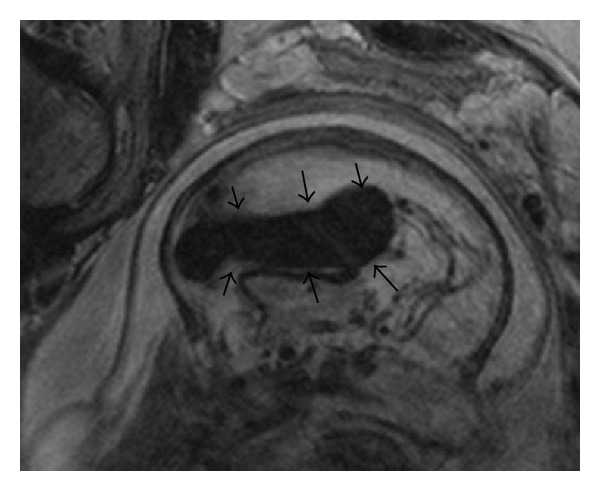
Fetal MRI imaging of aneurysm of vein of Galen.

As soon as ultrasound examination raised the possibility of a VGAM, an MRI was performed in order to thoroughly examine the fetal brain, heart, and chest. MRI confirmed the presence of an aneurysm of the vein of Galen (Figure 2). An elective caesarean section was performed at 33 weeks of gestation, and a male neonate weighing 2970 gm was born. The newborn was admitted to the Neonatal Intensive Care Unit. Full respiratory and medical support was given but the sustainment of cardiac failure resulted in neonatal death just one day following its admission.

## 3. Discussion

The management of the aneurysm of the vein of Galen is a great challenge, especially in case cardiac function is compromised at the time of diagnosis. Embolism is reported to be the optimal therapeutic strategy; however, it should be performed after stabilization of the neonate and preferably after the 5th or 6th month of life, with the exception of life-threatening conditions [6, 7]. Embolism is probably contraindicated when cardiomegaly or brain injury during labor is present [8]. Therefore, regarding complicated neonates with early development of severe heart dysfunction as in our case, vigorous respiratory and heart support in the Neonatal Intensive Care Unit is the first priority, keeping in mind that the prognosis is extremely poor [1].

Prenatal findings indicative of heart dysfunction are associated with a poor prognosis for the neonate. Sepulveda et al. [9], in a review of 23 cases with a diagnosis of an aneurysm of the vein of Galen, reported that in 10 of 13 cases with prenatally diagnosed cardiomegaly there was a neonatal or infant death. No information about long-term outcomes was available for the survivors. Frawley et al. [3] suggest that aggressive medical treatment and early neurointervention may contribute to improved clinical outcome even in cases in which severe heart dysfunction is already present at delivery.

Early detection of VGAM is crucial for optimal therapeutic approach. The diagnostic accuracy of ultrasound Doppler examination has already been advocated [10]. Rios et al. supported the usefulness of 3-diamensional Doppler assessment in the diagnostic approach of AVG as power Doppler has the capacity to obtain a signal in vessels with low-flow velocity and offers the possibility of a high-accuracy imaging reconstruction of vessels' architecture [11]. The contribution of MRI in the diagnosis of VGAM is advocated by Rodesch et al. [6] as it can assist in the early detection of VGAM or in the postnatal therapeutic management, offering the possibility of deciding on the optimal time for intervention throughout consecutive screening. In our case, the MRI was helpful in confirming the diagnosis of VGAM that was first suspected on ultrasound. 

In conclusion, we reported the case of vein of Galen with coexisting severe heart dysfunction diagnosed prenatally by ultrasound and MRI. Early detection of VGAM is important to maximize the chances of a favorable outcome in complicated cases with early development of severe heart dysfunction.
